# Health: A Poem, Shewing How to Procure, Preserve, and Restore It; to Which Is Added, the Doctor's Decade

**Published:** 1816-07

**Authors:** 


*Health: a Poem, shewing how to procure, preserve, and restore it; to which is added, the Doctor's Decade.* By EDWARD BAYNARD, M.D. pp. 32. 1816.

THE Muses have not disdained an acquaintance with Hygeia, nor even to sacrifice at the shrine of Cloacina, as the writings of Garth, Armstrong, and Darwin evince. Since the harmonious numbers of the last-mentioned author, Poetry has not, we believe, been seen in company with Physic; and, therefore, the publication of Edward Baynard, M. D. was no sooner proclaimed, than we ordered it from our bookseller, expecting an evening's relaxation from the dull monotony of *prosing* practice, and a pleasant excursion into the regions of fancy, from which we are now, alas, almost entirely excluded. For some days before the arrival of the poem, we had been brushing the dust off our classical and poetical ideas, (as a London Alderman whets his appetite with a little brandy before he sits down to the green fat of the turtle) in order that we might enjoy, with greater relish, the Parnassian banquet. The first couplet, however, of the Preface, gave a dreadful shock to the *Pierian* delicacy of our nerves.

> " Unerring Nature learn to follow close,
> " For *quantum sufficit* is just her *dose !*"

It has been somewhere observed, that human pleasure is all in *anticipation*, and we here felt the truth of the remark! Remembering, however, the other adage, that there is no book so bad, but some good may be gleaned from it; we persevered through this elegant production of Dr. Baynard; and if pleasure be a good, and laughter an *index* of pleasure, we certainly cannot complain that our half-crown has been entirely thrown away.

If our M. D's. similies are not exactly *homeric* in grandeur or sublimity, they sometimes possess a part of the sublime—mystery.

> " Trust not to *constitution*—'twill decay,
> " And twisted *strength* its fibres wear away.
> " As close wove *garments*, of a strong spun thread,
> " The *woof* frets out and tears away the *web ;*
> " So *soul* and *body*, tho' ne'er so well conjoined
> " The longer that they wear, the more they *grind.*"

Our author, however, is strictly orthodox in his doctrines and morality. He declaims with irresistible *eloquence* against women and wine—particularly the former.

> " In single life, live pure and chaste,
> " Lest from your face your *nose* you cast.
> " And is it not a great disgrace,
> " To lose the *bowsprit* of your face ?"

He is decidedly Anti-Brunonian, and we seriously recommend Dr. Baynard's doctrine to the perusal of some of our antiquated Medicos of the celebrated schools of debility, spasm, and putrescency, who may yet be seen in every town and village of Great Britain; and even in Berwick upon Tweed.

" Accustom early in your youth,

" To lay embargo on your *mouth;*

" To miss a *meal* is sometimes good,

" It ventilates and cools the blood,

" Gives Nature time to *clean her streets,*

" From filth and crudities of meats."

Dr. Baynard does not extend the purgative plan quite so far as some modern practitioners, and only has recourse to it in cases of great importance.

" But when the fevers *peracute,*

" It wont admit of long dispute—

" For then the case is very urging—

" Spare neither vomiting nor purging."

By this time our readers have had enough of Dr. Edward Baynard. We shall, therefore, close this article with an extract from this sapient M. D. respecting the circulation of the blood; recommending it to the attention of Drs. Parry and Carson, whose deductions on this subject, are so diametrically opposite.

" The heart *clacks* on, and is a *mill,*

" That's independent of the will;

" And like an *engine,* squirts the blood,

" Forcing *up hill,* the purple flood;

" A constant *fountain* that displays

" Its rivulets ten thousand ways.

" Its office is to *mesh* and *beat,*

" And make the chyle *assimilate!"*

How any member of a learned and dignified profession, and particularly a man attaching M. D. to his name, should dare to wilfully and maliciously degrade that profession, of which he is indeed the disgrace, by issuing to the public such wretched and abominable *stuff,* is to us surprising! All the punishment we can inflict, is to hold up the pamphlet to derision and contempt. All the apology we can offer for noticing it, is, that we ourselves were *taken in,* in the purchase and perusal of this contemptible performance.

